# The Resveratrol Tetramer (-)-Hopeaphenol Inhibits Type III Secretion in the Gram-Negative Pathogens *Yersinia pseudotuberculosis* and *Pseudomonas aeruginosa*

**DOI:** 10.1371/journal.pone.0081969

**Published:** 2013-12-04

**Authors:** Caroline E. Zetterström, Jenny Hasselgren, Olli Salin, Rohan A. Davis, Ronald J. Quinn, Charlotta Sundin, Mikael Elofsson

**Affiliations:** 1 Umeå Centre for Microbial Research, Laboratories for Infection Medicine Sweden, Department of Chemistry, Umeå University, Umeå, Sweden; 2 Creative Antibiotics Sweden AB, Umeå, Sweden; 3 Department of Clinical Microbiology, Umeå University, Umeå, Sweden; 4 Eskitis Institute, Griffith University, Brisbane, Australia; 5 Laboratories for Chemical Biology Umeå, Department of Chemistry, Umeå University, Umeå, Sweden; The Scripps Research Institute and Sorrento Therapeutics, Inc., United States of America

## Abstract

Society faces huge challenges, as a large number of bacteria have developed resistance towards many or all of the antibiotics currently available. Novel strategies that can help solve this problem are urgently needed. One such strategy is to target bacterial virulence, the ability to cause disease e.g., by inhibition of type III secretion systems (T3SSs) utilized by many clinically relevant gram-negative pathogens. Many of the antibiotics used today originate from natural sources. In contrast, most virulence-blocking compounds towards the T3SS identified so far are small organic molecules. A recent high-throughput screening of a prefractionated natural product library identified the resveratrol tetramer (-)-hopeaphenol as an inhibitor of the T3SS in *Yersinia pseudotuberculosis*. In this study we have investigated the virulence blocking properties of (-)-hopeaphenol in three different gram-negative bacteria. (-)-Hopeaphenol was found to have micromolar activity towards the T3SSs in *Yersinia pseudotuberculosis* and *Pseudomonas aeruginosa* in cell-based infection models. In addition (-)-hopeaphenol reduced cell entry and subsequent intracellular growth of *Chlamydia trachomatis*.

## Introduction

Over many years there has been an overuse of antibiotics to prevent and treat bacterial infections in humans and animals. As a result many bacterial strains have developed resistance towards many or even all of the antibiotics in current use. This is a serious problem all over the world and the need for novel therapies is urgent. One strategy for the discovery of novel antimicrobial compounds is to target the functions important for bacterial infection that cause disease, such as bacterial virulence [1]. By targeting virulence systems, the bacteria would be unarmed rather than killed and the reaction in the body would likely be similar to that following a vaccination with a live impaired bacterial strain. As bacteria would still be able to grow and proliferate, the innate and adaptive immune responses must clear the infection. One advantage of this novel approach is that the effect on the endogenous gut microflora would likely be minimal since these beneficial microbes lack virulence systems like the type III secretion system (T3SS). It is hypothesized that the selective pressure to develop resistance towards these kinds of compounds is subdued compared to conventional antibiotics [1-3]. Virulence-blocking compounds could be designed to target many different mechanisms e.g. adhesion to the target cell [4], bacterial signaling [5] or toxin delivery systems, such as the T3SS [6]. The T3SS is an attractive drug target since it is a conserved virulence system utilized by many gram-negative animal and plant pathogens such as *Yersinia* spp., *Pseudomonas aeruginosa, Chlamydia* spp., *Salmonella* spp., *Shigella* spp, enteropathogenic *Escherichia coli* (EPEC) enterohemorrhagic *E. coli* (EHEC), and *Erwinia* spp. [1,7]. The bacteria use the T3SS to secrete and translocate different effector proteins into the eukaryotic host cell cytosol. *Yersiniae* have six different effector proteins known as Yops (*Yersinia* outer proteins) some of which are essential for virulence. The effector proteins work in ensemble inhibiting innate immunity and destroying the cytoskeleton in the host cell, thereby allowing the bacteria to avoid phagocytosis, proliferate and spread to new host [8]. *Chlamydiae* are likely to use the T3SS to facilitate their entry into host cells and to inject effector proteins through the inclusion membrane, surrounding the intracellular bacteria, to the host cytosol [9]. 

In the last ten years many different synthetic virulence-blocking compounds targeting different T3SSs have been identified mainly using screening-based approaches [10-21] and the field has been extensively reviewed [22-24]. The *Yersinia* genus consists of eleven species, three of which is pathogenic to man i.e., *Y. entercolitica*, *Y. pestis*, and *Y. pseudotuberculosis* [8]. *Y. pestis* is probably the most well-known since it caused the bubonic plague/Black Death in the middle of the 14^th^ century. *Y. entercolitica* and *Y. pseudotuberculosis* cause inflammation in the gastrointestinal tract of humans and spread through the fecal-oral route, usually from contaminated food or water. In one of our earlier reports we screened for T3SS inhibitors using *Y. pseudotuberculosis* as the model organism since its T3SS is well studied and can be manipulated *in vitro* [6]. Important features are that assembly of the T3SS can be induced by a temperature shift from 26 °C to 37 °C, and that secretion can be triggered by removal of calcium without requirement for host cell contact. We have previously identified three compound classes: the salicylanilides, the 2-arylsulfonylamino-benzanilides, and the salicylidene acylhydrazides as T3SS inhibitors that blocked secretion of the effector proteins in *Y. pseudotuberculosis* [6]. These compound classes were further explored with design, synthesis and biological evaluation of analogs. The biological data were then successfully linked to the chemical structure with quantitative structure-activity relationships (QSARs) [25-29]. The salicylidene acylhydrazides target the T3SSs in several pathogens [22-24] and we recently concluded that the salicylidene acylhydrazides most likely interact with multiple proteins, several of which are involved in cell metabolism, with down regulation of T3SS functions as a net result [30]. The compounds have also proven efficacious in a *Chlamydia trachomatis* vaginal mouse infection model and thus indicate that the T3SS is a validated drug target [31,32]. Our conclusions, taken together with other researchers findings support the hypothesis that the T3SSs are validated targets for the development of small molecule drugs. These virulence-blocking agents may become invaluable for the prevention or treatment of bacterial infections either as stand-alone therapeutics or as adjuncts to conventional antibiotics.

Most of the T3SS inhibitors described in the literature are synthetic small organic and drug-like molecules. Considering that the majority of the successful antibiotics in use today are of natural origin it is tempting to speculate that it should be possible to identify T3SS inhibitors from natural sources e.g. plants, microorganisms, and invertebrates. A few studies indicate that nature indeed can furnish T3SS blocking compounds. In 2002 screening of marine invertebrate extracts identified caminoside A as an inhibitor for T3SS in EPEC [33]. Later caminocide B-D were also found to be T3SS inhibitors [34]. Interestingly, the compounds were also shown to have antimicrobial activity against vancomycin resistant *Enterococcus*, as well as against methicillin resistant *Staphylococcus aureus*, suggesting that the caminosides might act as general antibiotics rather than selective T3SS inhibitors [33,34]. In another study, extraction of a *Streptomyces* broth (soil-derived sp.) furnished guadinimine A-F that inhibited induced T3SS dependent hemolysis of erythrocytes by EPEC [35]. The data suggest that the guadinimines are potent natural product inhibitors of the T3SS in EPEC [35,36]. Aurodox is another natural product found in an extract from an *Actinomycete* strain and it was recently shown to inhibit EPEC T3SS-mediated hemolysis. The compound also proved efficacious in an *in vivo* study where mice were infected with a lethal dose of *Citrobacter rodentium* [37]. Aurodox is a known antibiotic that inhibits protein biosynthesis specifically by binding to bacterial elongation factor Tu [38] however, the mechanism for its T3SS inhibition remains unclear.

Our collaboration has previously reported the identification and total synthesis of pseudoceramines A-D and spermatinamine as putative inhibitors of T3SS in *Y. pseudotuberculosis* from high-throughput screening of the Eskitis Nature Bank prefractionated natural product library [39-41]. These compounds were identified using a primary reporter-gene assay with luciferase under control of the YopE promoter and subsequent counter screens based on the phosphatase activity of secreted YopH which have been described previously [6,39]. The compounds were derived from a marine sponge *Pseudoceratina* sp. and pseudoceramine B and spermatinamine inhibited the T3SS system in the low micromolar range. In addition to the attenuation of T3SS, the compounds also exhibited an effect on bacterial growth, indicating general antibacterial properties [39]. 

**Figure 1:**
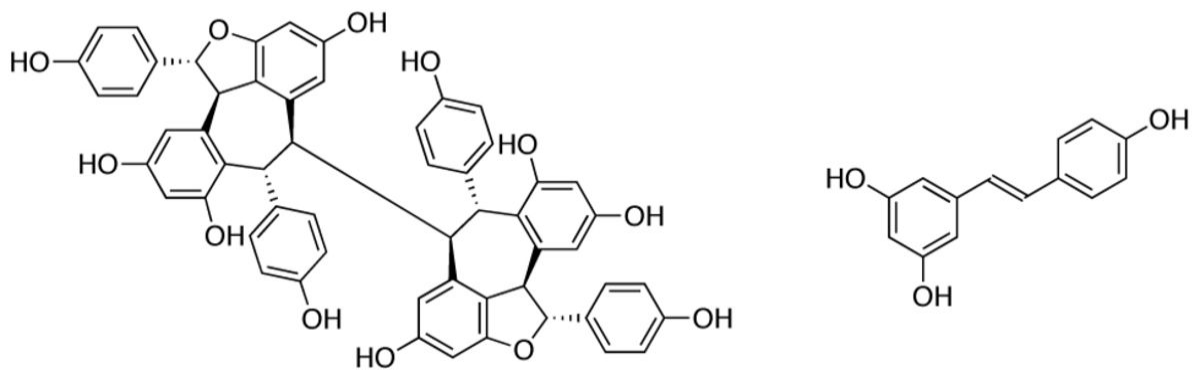
Structure of (-)-hopeaphenol and resveratrol.

In the same screening campaign bioassay-guided fractionation on the leaf extracts derived from two Papua New Guinean rainforest plants, *Anisoptera thurifera* and *A. polyandra*, identified (-)-hopeaphenol (Figure 1) together with other less active stilbenoids as putative T3SS inhibitors. The elucidation of (-)-hopeaphenol [42], a complex tetrameric stilbenoid was achieved by spectroscopic and crystallographic methods. A full disclosure of the screening, isolation and characterization of (-)-hopeaphenol will be described elsewhere. The compound belongs to the hopeaphenol class of polyphenols, which are all tetramers of resveratrol: a well-studied trans-stilbene containing two hydroxylated phenyls connected with a two-carbon methylene bridge (Figure 1). The first biological activity of resveratrol was published 1992 [43] and since then it has been shown to have anticancer, antifungal, anti-inflammatory and antimicrobial properties [44,45]. In nature resveratrol is a common building block used to assemble, presumably via oxidative coupling reactions, a large number of natural products with varying complexity in terms of scaffold structures and stereochemistry that often are further diversified by e.g. glycosylation [45]. Despite their complexity a few natural products based on resveratrol oligomers have been achieved [46-48]. In this study we investigated the effect of (-)-hopeaphenol on the T3SSs in *Y. pseudotuberculosis*, *P. aeruginosa* and *C. trachomatis*. 

## Results and Discussion

###  (-)-Hopeaphenol inhibits YopE expression and YopH secretion

**Figure 2:**
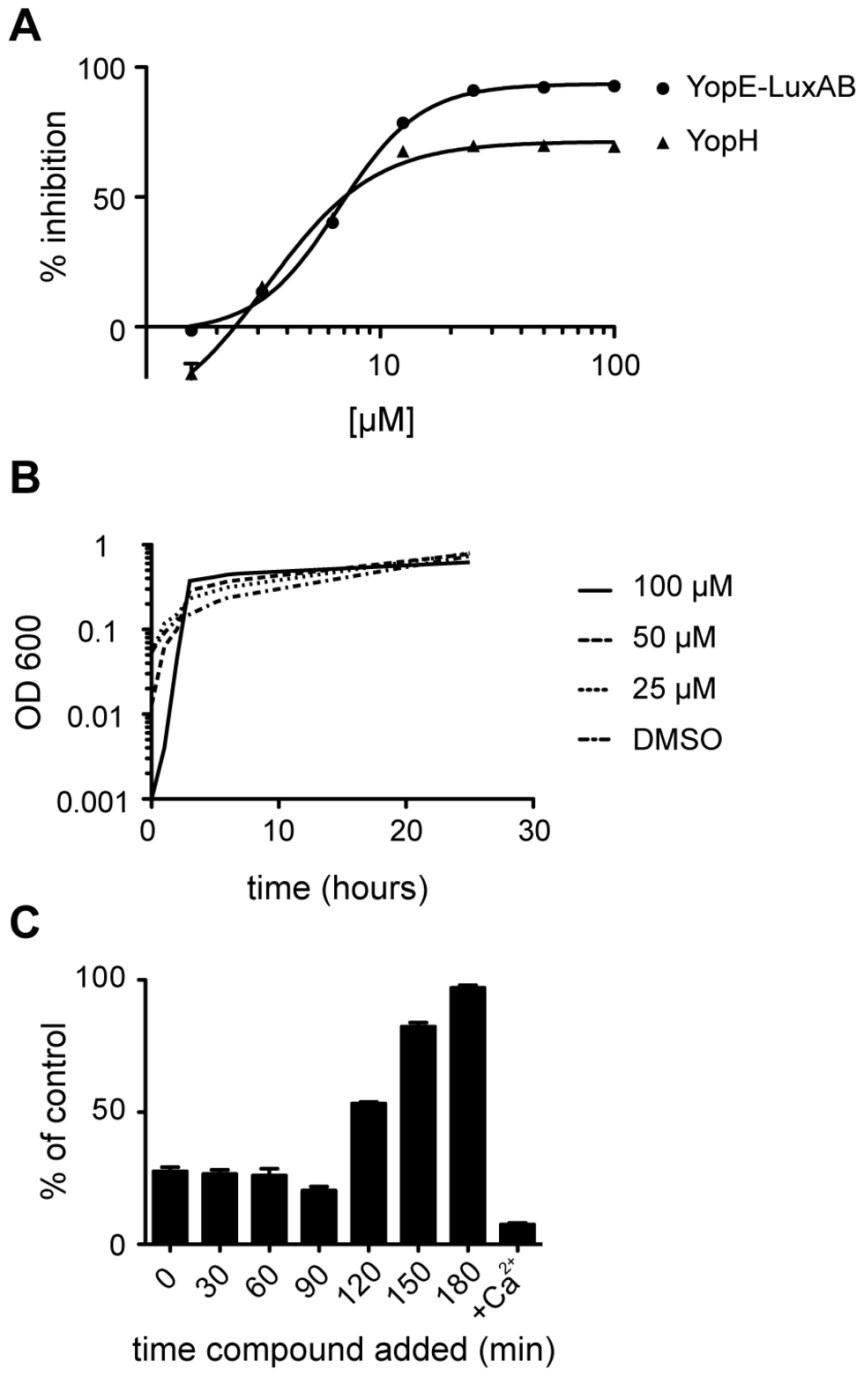
(-)-Hopeaphenol inhibits the effector protein secretion in *Y. pseudotuberculosis* but does not affect growth. (A) Circles show inhibition of YopE and the luciferase light signal with an IC_50_ of 6.6 µM. Triangles show inhibition of the enzymatic signal from YopH in *Y. pseudotuberculosis*. (B) Growth inhibition curve of *Y. pseudotuberculosis* treated with three different concentrations of (-)-hopeaphenol. (C) Time study of the YopE and the luciferase light signal in *Y. pseudotuberculosis*.

As a first step we confirmed the activity of (-)-hopeaphenol in the primary screening YopE reporter-gene assay and the YopH phosphatase assay. Figure 2A shows the dose-response curves from the reporter-gene and phosphatase assays with IC_50_ values of 6.6 µM and 3.3 µM, respectively. (-)-Hopeaphenol had no or limited effect on bacterial growth at concentrations up to 100 µM (Figure 2B). To establish if addition of (-)-hopeaphenol at different stages of the induction and secretion phases of *Y. pseudotuberculosis* T3SS affect the efficacy the compound was added at seven time points during infectious conditions. The compound (50 µM) was added every 30th min for 3 h and the temperature shift from 26 to 37 °C was made at *t* = 60 min, mimicking eukaryote cell contact that trigger effector protein secretion and translocation. Addition of (-)-hopeaphenol up to *t* = 90 min strongly attenuated the reporter-gene signal while addition at later time points resulted in a diminished effect (Figure 2C). This indicated that the effect of (-)-hopeaphenol was rapid, similar to previously reported data for salicylidene acylhydrazides in general, and ME0052/INP0010 in particular [26,29]. In this study ME0052/INP0010 was used as a control, and gave the same result as described previously (data not shown) [26]. 

### Dose-dependent inhibition of expression and secretion of the translocator protein YopD

**Figure 3:**
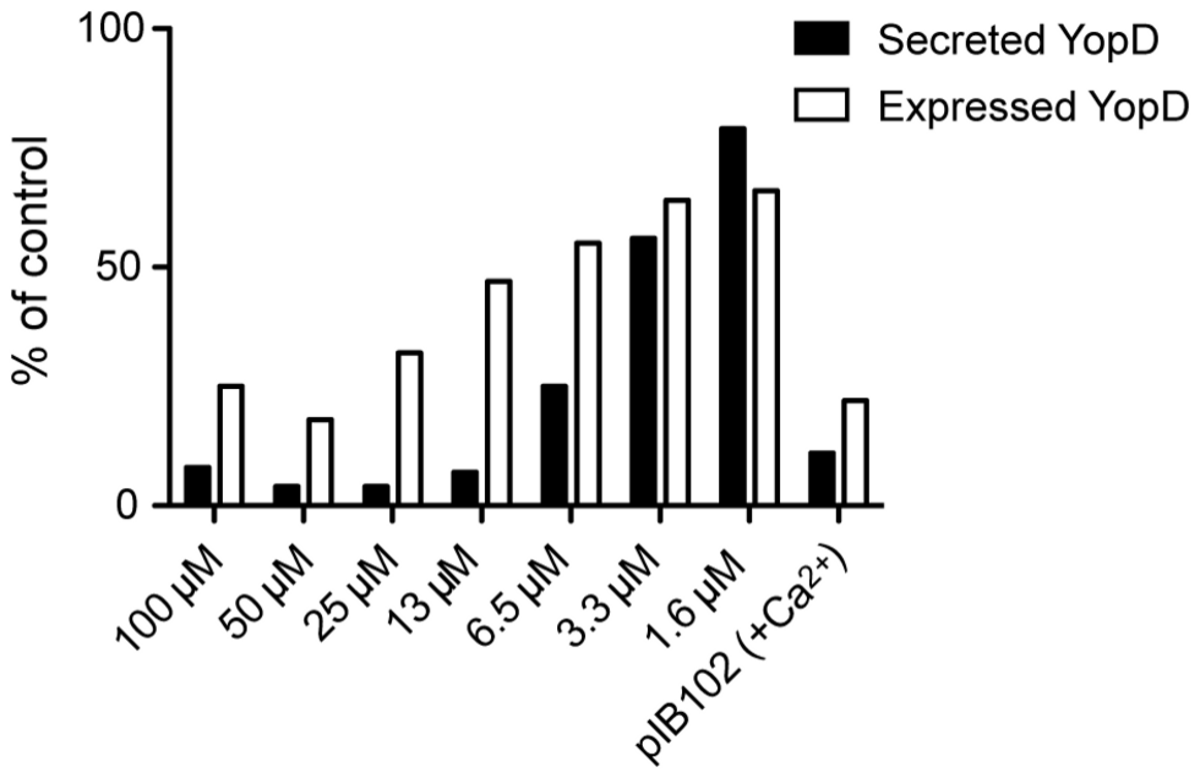
Western analysis on *Y. pseudotuberculosis* treated with (-)-hopeaphenol. The T3SS was induced in *Y. pseudotuberculosis* wild-type strain treated with (-)-hopeaphenol concentrations from 1.6 to 100 µM. Western analysis was performed on secreted and expressed YopD and compared to DMSO control.

Western blot analysis of effector proteins in total culture and supernatant, of YPIII(pIB102) wild-type bacteria incubated together with 7 different concentrations of (-)-hopeaphenol for 1 h at 26 °C followed by 3 h at 37 °C showed a clear dose-dependent response for both secretion and expression of translocator protein YopD (Figure 3). The expression of YopD was reduced but could be detected at all concentrations, whereas concentrations above 13 µM completely blocked the secretion of YopD. These results are in line with the data obtained from the YopE reporter-gene and YopH phosphatase assays (Figure 2A), suggesting that (-)-hopeaphenol might target the secretion machinery directly rather than a general attenuation T3SS genes transcription as observed for the salicylidene acylhydrazides in *E. coli* [49]. 

### (-)-Hopeaphenol is an irreversible T3SS inhibitor

**Figure 4:**
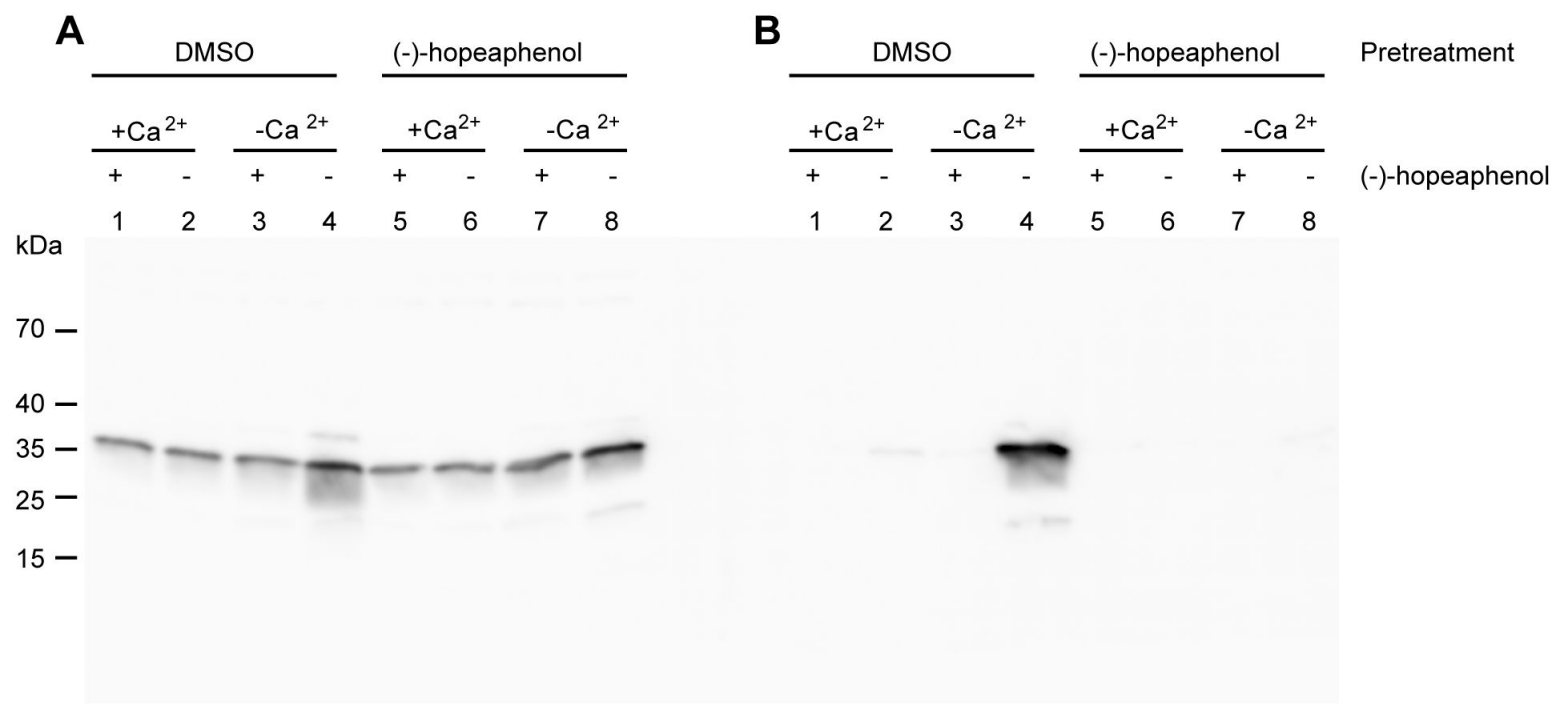
The effect of (-)-hopeaphenol treated *Y. pseudotuberculosis* is irreversible. Western blot analysis of the reversibility of (-)-hopeaphenol treatment of *Y. pseudotuberculosis*. Lanes 1-4 have been pretreated with DMSO and lanes 5-8 with (-)-hopeaphenol. After pretreatment the two cultures were divided into four. Out of the four, two were diluted in LB supplemented with Ca^2+^ and the other two in Ca^2+^ depleted LB. (-)-Hopeaphenol (40 µM final concentration) or DMSO were then added to the different conditions. (A) Total culture. (B) Supernatant.

To investigate if the effect of (-)-hopeaphenol on the T3SS is reversible, overnight cultures of YPIII(pIB102) were diluted and divided into two cultures that were treated with either (-)-hopeaphenol (40 µM) or DMSO alone. To fully induce the T3SS without triggering effector protein secretion, the cultures were grown in calcium containing medium for 30 min at 26 °C followed by 2 h at 37 °C. The cultures were then divided into eight tubes, with or without 40 µM (-)-hopeaphenol. The cultures were incubated at 37 °C for an additional 45 min to trigger effector protein secretion. Western blot analysis was performed on the total culture and the supernatant. The total culture samples all showed Yop expression indicating that (-)-hopeaphenol inhibited secretion but not expression (Figure 4A, lane 1-8). None of the cultures grown in the presence of calcium secreted any Yops irrespective of the pretreatment conditions (Figure 4B, lane 1, 3, 5, and 7). The culture pretreated with DMSO grown in calcium depletion secretes Yops into the media (Figure 4B, lane 4). When (-)-hopeaphenol was added to the culture pretreated with DMSO in presence of calcium secretion was completely blocked (Figure 4B, lane 2). This result shows that the effect of (-)-hopeaphenol on the T3SS is rapid. The cultures pretreated with (-)-hopeaphenol were unable to secrete effector proteins both in the DMSO control and when treated with (-)-hopeaphenol independent of the presence or absence of calcium (Figure 4B, lane 5-8). Thus, the effect of (-)-hopeaphenol is not reversible and the results suggest that the compound might bind covalently to its target(s) and that the targets possibly are located on the bacterial cell surface e.g. the extracellular structure of the T3SS. 

### (-)-Hopeaphenol inhibits YopE translocation

**Figure 5:**
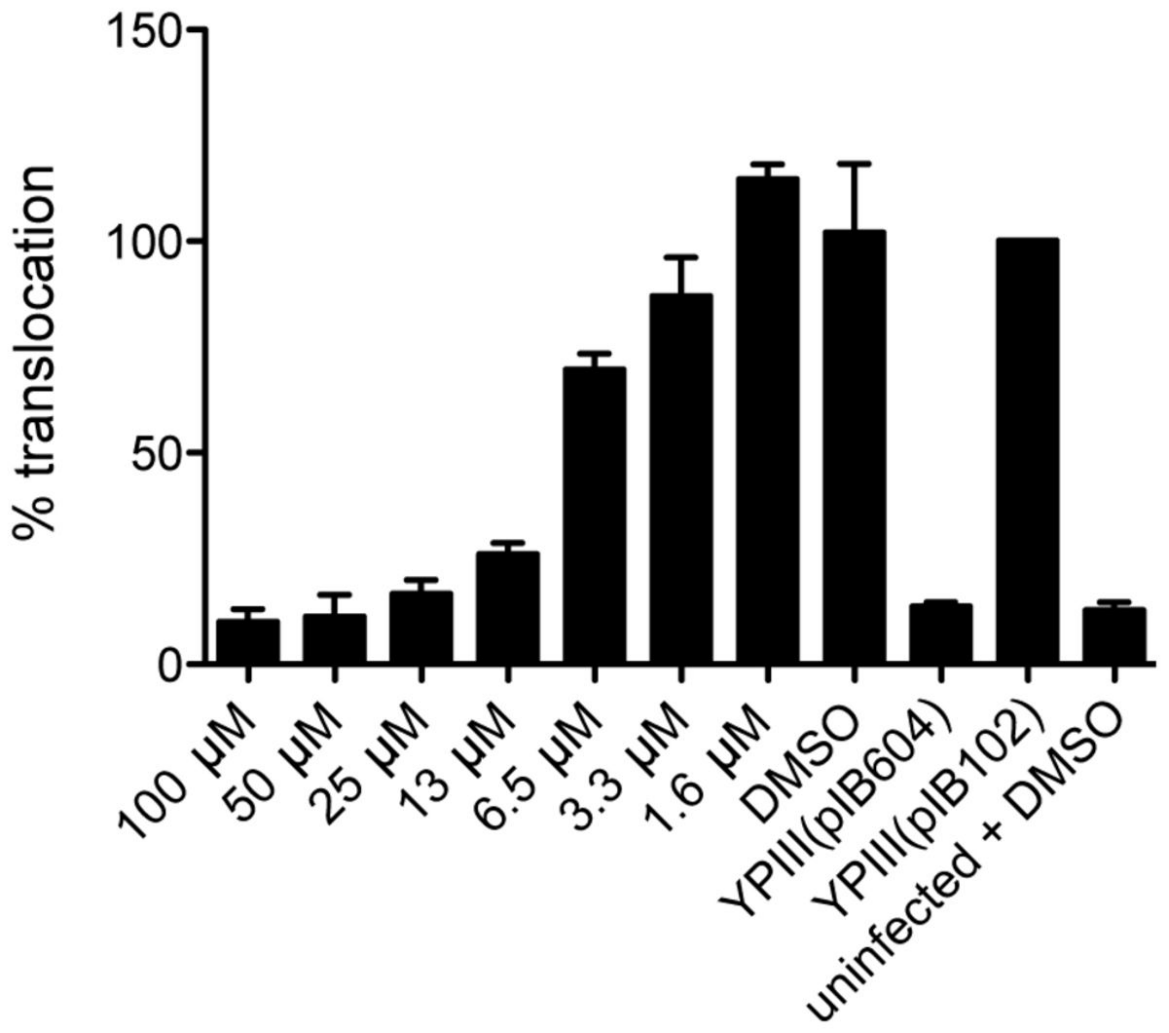
(-)-Hopeaphenol affects translocation by *Y. pseudotuberculosis* in a dose dependent manner. HeLa cells were infected for one hour with *Y. pseudotuberculosis* treated with (-)-hopeaphenol concentrations between 1.6 - 100 µM. Controls cells are infected with YPIII(pIB604) Δ*yopB* a translocation deficient mutant, wild-type YPIII(pIB102) and left uninfected with DMSO.

**Figure 6:**
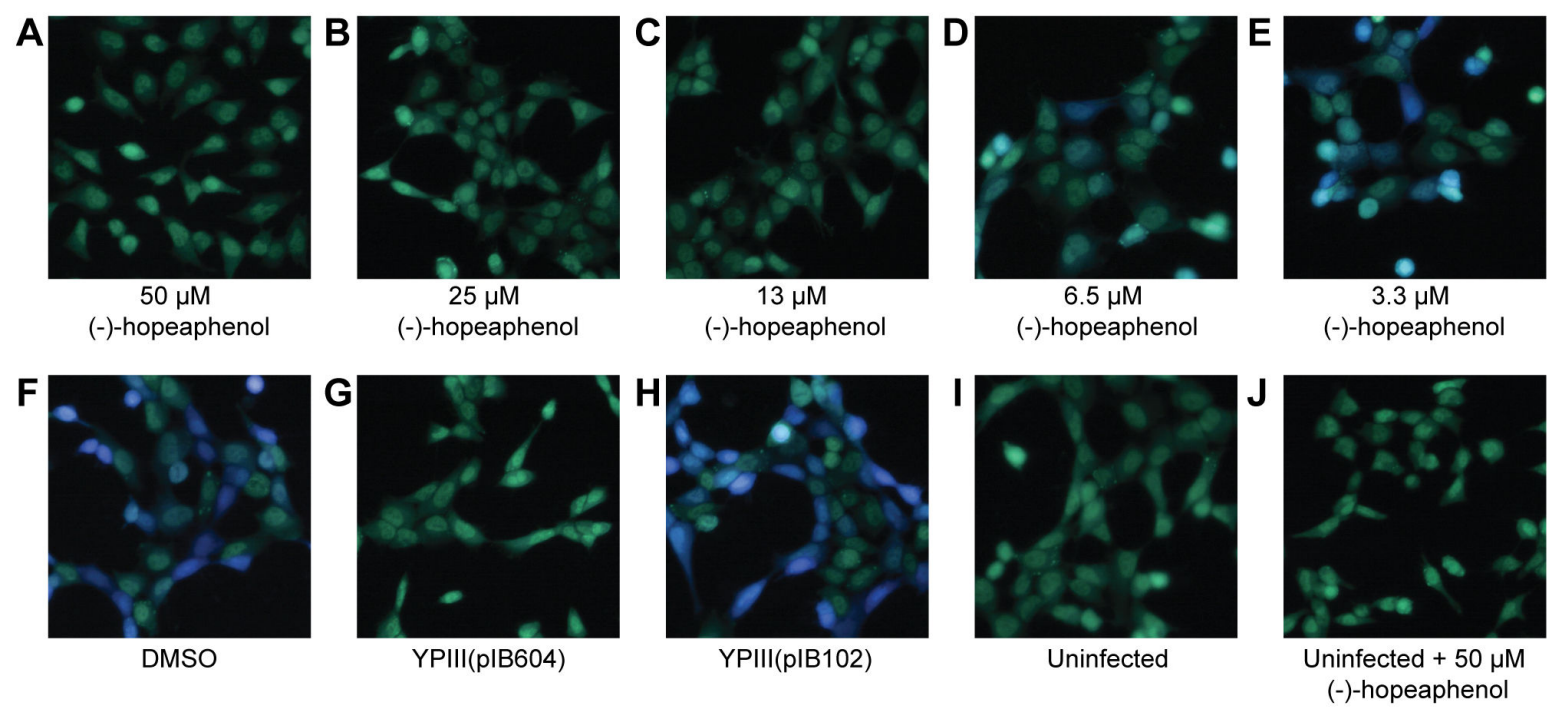
(-)-Hopeaphenol inhibits translocation of effector proteins from *Y. pseudotuberculosis* into HeLa cells. (A-F) HeLa cells infected with wild-type YPIII(pIB102) bacteria with or without (-)-hopeaphenol 3.3-50 µM or DMSO alone. (G-J) Controls cells were infected with YPIII(pIB604) Δ*yopB* a translocation deficient mutant, wild-type YPIII(pIB102) and left uninfected with or without 50 µM (-)-hopeaphenol.

To investigate if (-)-hopeaphenol also inhibits *Y. pseudotuberculosis* effector protein translocation and thereby virulence, a β-lactamase reporter system was used [50-52]. The bacterial strains used, have the effector protein YopE translationally fused to β-lactamase. HeLa cells were infected with YPIII(pIB102) *yopE-bla* with a multiplicity of infection (MOI) of 50 times and (-)-hopeaphenol was added at different concentrations. As a control experiment HeLa cells were also infected with a translocation deficient mutant YPIII(pIB604) Δ*yopB yopE-bla*. After infection the HeLa cells were loaded with CCF4-AM, which consists of a cephalosporin core linking 7-hydroxycoumarine to a fluorescein. Inside the cells the ester of the substrate will be hydrolyzed to its negatively charged form CCF4, and retained in the cytosol. In the absence of β-lactamase, excitation of the coumarine at 409 nm will result in a Förster resonance energy transfer (FRET) and the emitted light at 520 nm can be detected as a green fluorescent signal. This was the case for the HeLa cells where no translocation of YopE-Bla has occurred. When translocation has occurred the β-lactamase will act on cephalosporin and cleave the substrate resulting in disruption of the FRET, which after excitation at 409 nm result in light emitted at 450 nm, a blue light signal. The light signal was measured in a microplate reader and percentage of translocation, the blue light signal, was calculated and normalized to DMSO treated YPIII(pIB102) wild-type set to 100 % translocation. The infected HeLa cells were analyzed with fluorescent microscopy as a supplement to the microplate reader data. Treatment with (-)-hopeaphenol resulted in a dose-dependent response (Figure 5 and Figure 6A-C). (-)-Hopeaphenol inhibits translocation of YopE completely at 50, 25, and 13 µM (Figure 5 and 6A-C) and at lower concentrations translocation could be observed (Figure 5 and 6D-E). Infected cells treated with or without DMSO as control have the same amount of translocated YopE-Bla (Figure 6F and 6H). As additional controls cells were infected with YPIII(pIB604) Δ*yopB* (Figure 6G), a translocation deficient mutant, YPIII(pIB102) wild-type (Figure 6H) and left uninfected, with or without 50 µM (-)-hopeaphenol (Figure 6I and 6J). 

### (-)-Hopeaphenol blocks ExoS expression and secretion by *P. aeruginosa*

**Figure 7:**
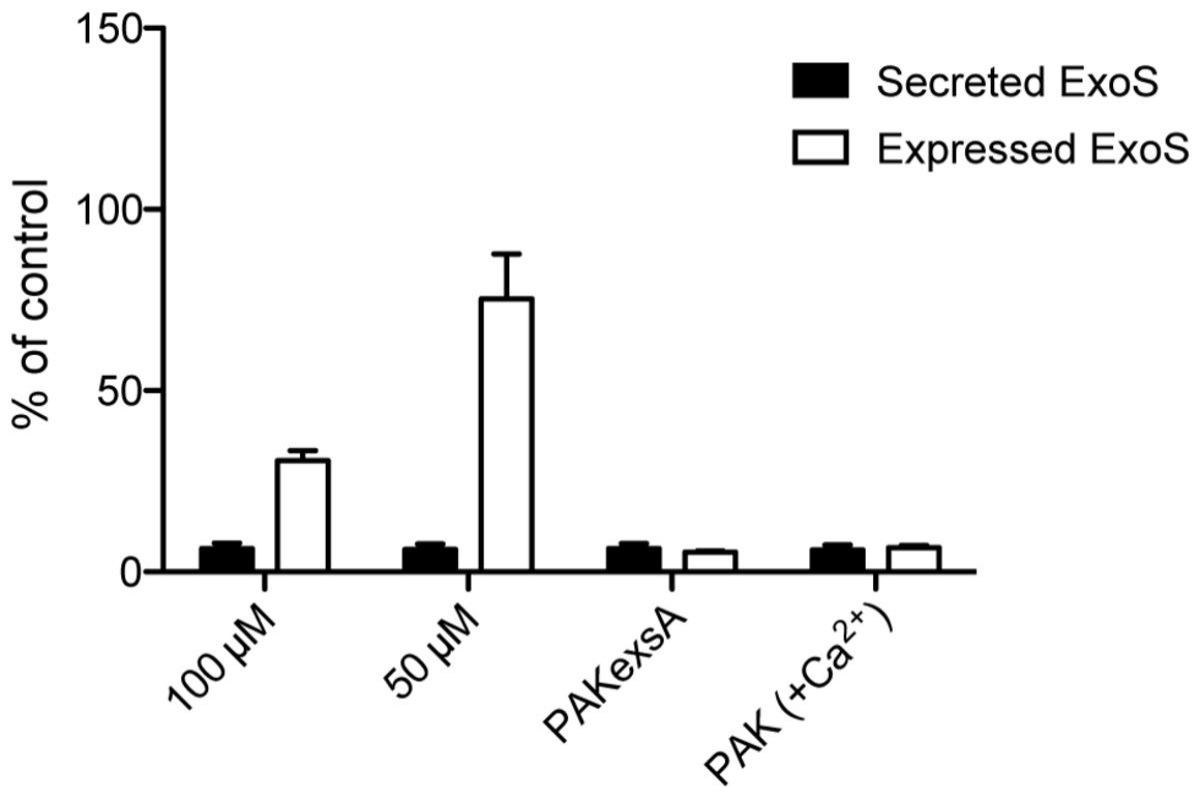
(-)-Hopeaphenol inhibits secretion and expression of the *P. aeruginosa* T3SS effector protein ExoS. The T3SS was induced in wild-type *P. aeruginosa*, strain PAK, by removal of Ca^2+^ from the medium and (-)-hopeaphenol was added. After 3 hours of incubation the secretion and expression of the T3SS effector protein ExoS was measured by western analysis and compared to the DMSO control.

We decided to examine if (-)-hopeaphenol was able to inhibit the T3SS in *Pseudomonas aeruginosa* a gram-negative pathogen with a T3SS system that is closely related to the T3SS of *Y. pseudotuberculosis. P. aeruginosa* is an opportunistic human pathogen infecting burn wounds, as well as immunocompromised, and leukemia patients [53]. It belongs to the six ESKAPE pathogens (*Enterococcus faecium, Staphylococcus aureus, Klebsiella pneumoniae, Acinetobacter* *species, P. aeruginosa, and Entero-bacter species*). Together with *Mycobacterium tuberculosis* they are recognized as the most significant emerging threats of this century [54]. *P. aeruginosa* is a “superbug” with a unique capacity to develop resistance due to a combination of intrinsic, acquired and adaptive mechanisms [55]. As a consequence, today we have multi-drug resistant *P. aeruginosa* strains for which there are no effective antibiotic treatments available and the trend is that these strains are becoming more common. The need for new antimicrobial drugs for treatment of these multi-resistant strains is extremely urgent and should not be underestimated. The effector protein ExoS from *P. aeruginosa* is highly similar to the *Y. pseudotuberculosis* effector protein YopE [56] and as with *Yersinia* the T3SS can be triggered by growth at 37 °C and removal of calcium. The wild-type *P. aeruginosa*, strain PAK was grown in calcium-depleted media with or without (-)-hopeaphenol. Western blot analysis of the effector protein ExoS showed that the amount expressed and secreted ExoS was reduced in bacteria treated with (-)-hopeaphenol compared to the DMSO control. Secretion of ExoS is essentially completely blocked by (-)-hopeaphenol at 50 and 100 µM (Figure 7), while treatment with 10 and 20 µM (-)-hopeaphenol reduced the secretion. Inhibition of ExoS expression is dose-dependent as ExoS levels are higher in the bacteria treated with 10 µM (-)-hopeaphenol compared to bacteria treated with 100 µM (Figure 7). 

### (-)-Hopeaphenol inhibits *P. aeruginosa* virulence

**Figure 8:**
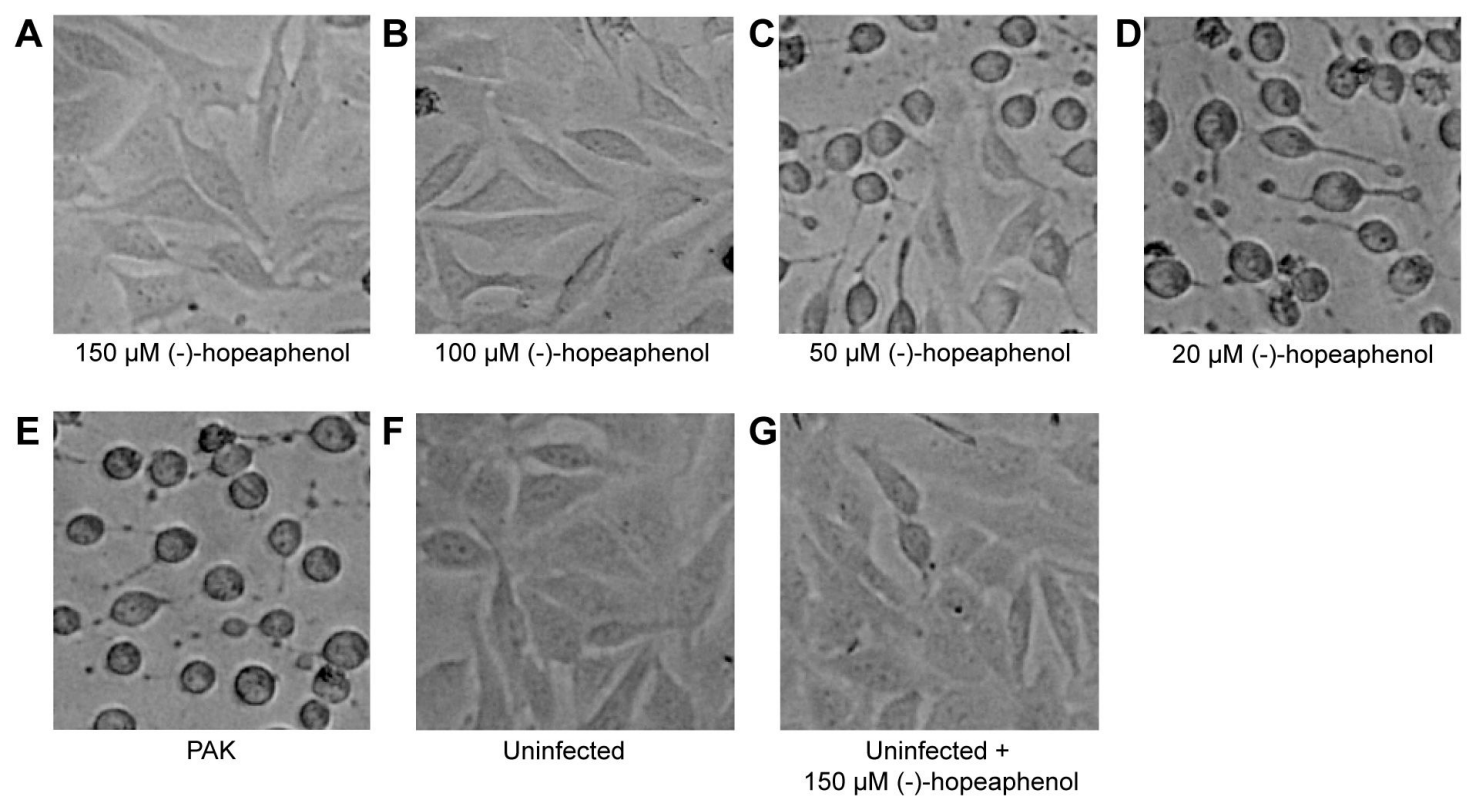
(-)-Hopeaphenol inhibits the infection of *P. aeruginosa* in HeLa cells. (A-D) HeLa cells infected with wild-type *P. aeruginosa*, strain PAK for 5 h with (-)-hopeaphenol concentrations ranging from 150 to 20 µM. (E-G) Controls with untreated infection, uninfected cells and uninfected cells with addition of 150 µM (-)-hopeaphenol.

To investigate if (-)-hopeaphenol could inhibit the virulence of *P. aeruginosa*, HeLa cells were infected for 5 h with the wild-type *P. aeruginosa*, strain PAK in the presence of (-)-hopeaphenol at four different concentrations ranging from 20-150 µM. Infected cells treated with 100 and 150 µM (-)-hopeaphenol were completely protected from infection (Figure 8A and 8B), whereas treatment with 50 µM cells were partly protected (Figure 8C). When cells were treated with 20 µM (-)-hopeaphenol, inhibition of virulence could not be observed (Figure 8D). Uninfected cells treated with 150 µM (-)-hopeaphenol for 5 h did not show any signs of toxicity. The results from our experiments with *Y. pseudotuberculosis* and *P. aeruginosa* corroborate each other and support the notion that (-)-hopeaphenol is a T3SS inhibitor. 

### (-)-Hopeaphenol prevents cell entry and intracellular growth of *C. trachomatis*

**Figure 9:**
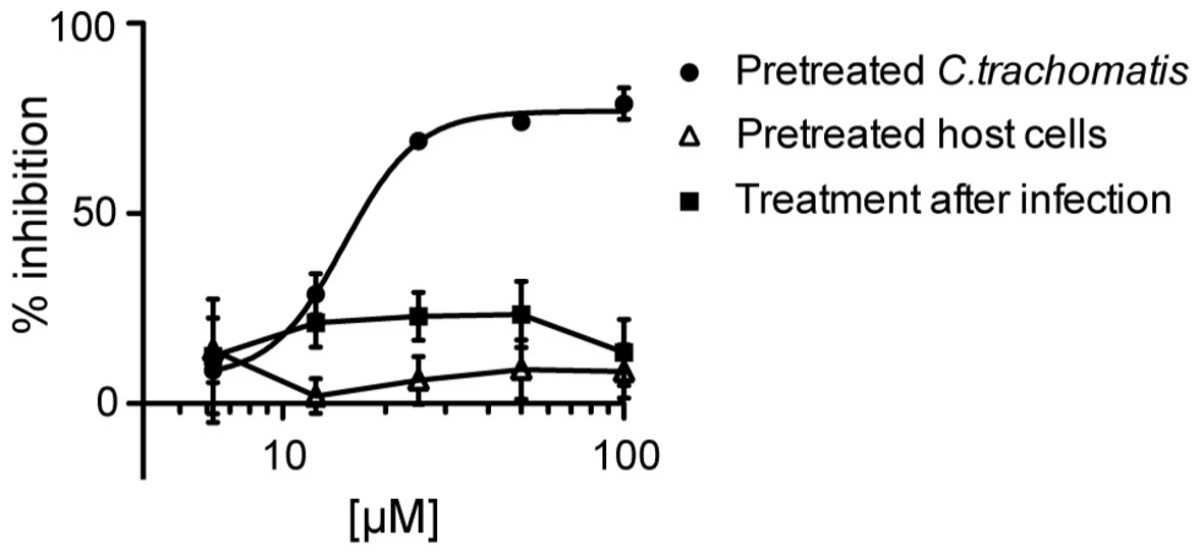
*C. trachomatis* pretreated with (-)-hopeaphenol. Circles, dose dependent response of *C. trachomatis* pretreated for 1 h with 6.5 – 100 µM (-)-hopeaphenol. Open triangles, HeLa cells pretreated with equal concentrations of (-)-hopeaphenol. Squares, treatment of *C. trachomatis* with equal concentrations (-)-hopeaphenol was made 1 h after infection.

**Figure 10:**
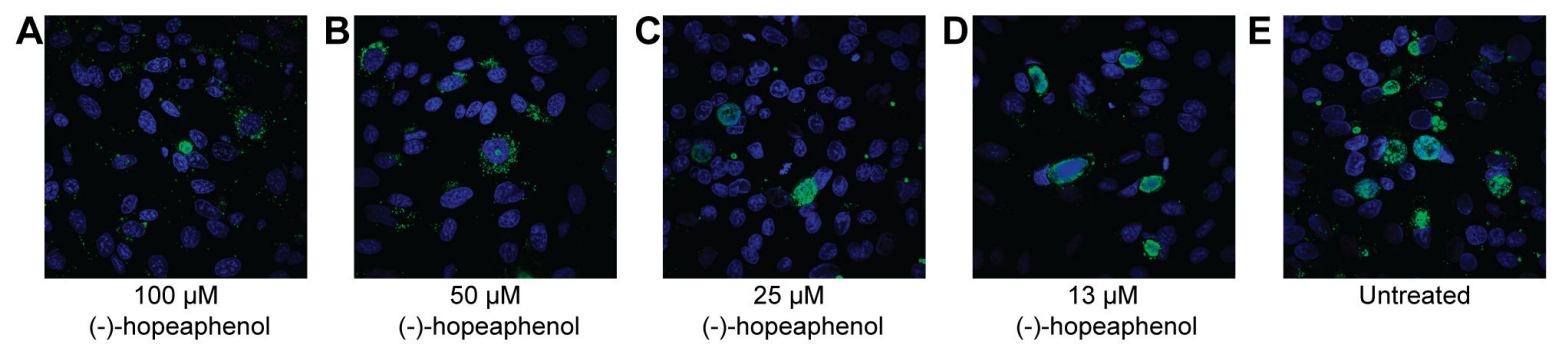
(-)-Hopeaphenol inhibits growth of *C. trachomatis* in HeLa cells. (A-D) HeLa cells infected with (-)-hopeaphenol pretreated *C. trachomatis* for 48 h. (-)-Hopeaphenol concentrations ranging from 100 to 13 µM. (E) Control pretreated with DMSO.

Previously, we have shown that the salicylidene acylhydrazides block the T3SSs in a number of pathogens: *Chlamydia trachomatis* [57-60]; *Chlamydia pneumonia* [60]; *Salmonella enterica* [61]; *Shigella flexneri* [62]; EHEC/EPEC [49] and most recently *Erwinia amylovora* [63]. The pathogens all have T3SSs but infect different hosts, show varying tropism, and cause a wide variety of diseases. *Chlamydia* is an obligate intracellular pathogen that can only grow inside a eukaryotic cell. *Chlamydiae* have a very different life cycle compared to both *Yersinia* and *Pseudomonas* and the role of the T3SS in *Chlamydia* is still enigmatic. It has a biphasic life cycle consisting of two distinct forms, the infectious elementary body (EB) and the replicative reticulate body (RB). *C. trachomatis* is mostly known to causes the sexually transmitted disease but it can also cause eye infections. It has been stated that T3SS is most likely essential for the survival of *Chlamydia* [9], and we therefore we tested the effect of (-)-hopeaphenol on *C. trachomatis* growth. HeLa cells were infected with *C. trachomatis* EBs and treated with five different concentrations, ranging between 6 and 100 µM of (-)-hopeaphenol 1 h after infection as per the standard procedure. After 19 h infected cells and *C. trrachomatis* inclusions were stained and analyzed with an automated microscope. However we could not observe any effect on *C. trachomatis* growth when treated with natural product at concentrations up to 100 µM (Figure 9). Since (-)-hopeaphenol is large with many polar hydroxyl groups it probably has low permeability both in eukaryotic and prokaryotic cells. The *Chlamydia* inclusion have low permeability for compounds larger than 520 Da [64], and we therefore decided to pretreat the bacteria with (-)-hopeaphenol before infection of host cells in order to address if the compound can prevent cell entry. *C. trachomatis* EBs were pretreated with different concentrations of (-)-hopeaphenol for 1 h followed by washing and infection of HeLa cells. Analyses of this experimental setup show that (-)-hopeaphenol inhibit infection and growth of *C. trachomatis* with a clear dose-response pattern (Figure 9 and 10). Pretreatment of the HeLa cells with (-)-hopeaphenol resulted in little or no effect on the *Chlamydia* infection, indicating that the compound directly targets *Chlamydia* (Figure 9). Altogether these results indicate that the permeability of (-)-hopeaphenol is low and we hypothesize that (-)-hopeaphenol binds covalent to *C. trachomatis* EBs, and thereby prevents cell entry and intracellular replication. These data correspond with the data showing an irreversible effect in *Y. pseudotuberculosis*. 

### (-)-Hopeaphenol does not affect growth of a panel of gram-positive and gram-negative pathogens

Finally a panel of five gram-positive and five gram-negative bacteria were tested for growth inhibition by (-)-hopeaphenol. The gram-positive bacteria tested were: *Micrococcus luteus, Staphylococcus epidermis, Staphylococcus aeureus, Bacillus subtilis*, and *Enterococcus faecalis* and the gram-negative bacteria tested (in addition to *Y. pseudotuberculosis* and *P. aeruginosa*) were *Proteus mirabilis, Klebsiella pneumonia*, and *E. coli K12*. None of the strains were significantly affected when grown together with 25, 50, or 100 µM (-)-hopeaphenol for 24 h (data not shown). The data suggest that (-)-hopeaphenol is a selective inhibitor of the T3SS in *Y. pseudotuberculosis* and *P. aeruginosa.*

## Conclusion

In summary, (-)-hopeaphenol was found to act as a T3SS inhibitor with an IC_50_ value of 6.6 µM in the YopE reporter-gene assay. Western blot analysis indicated dose-dependent inhibition of secretion with less reduction of Yop expression. Complete inhibition of Yop secretion occurred at concentrations that do not cause any detrimental effect on bacterial growth, indicating a selective inhibition of the T3SS. This is further corroborated by lack of growth inhibition in a panel of gram-positive and gram-negative bacteria. Interestingly pretreatment and washout experiments with (-)-hopeaphenol showed that the compound is an irreversible inhibitor of Yop secretion and suggest that the compound possibly binds covalently to its target(s). Considering the size and molecular weight of (-)-hopeaphenol cell permeability might be low which in turn suggest that inhibition occurs by interaction of T3SS components on the bacterial surface. However, no predictive models for permeability of bacteria exist and many antibiotics are typically larger and more hydrophilic than other types of drugs. We also identified that (-)-hopeaphenol inhibits the T3SS in the clinically challenging pathogen *P. auerginosa*. In *ex vivo* infection models using HeLa cells the compound blocks translocation of effector proteins by *Y. pseudotuberculosis* and prevents cytotoxicity caused by *P. aeruginosa*. In addition, we have showed that (-)-hopeaphenol inhibits growth of the intracellular pathogen *C. trachomatis* when pretreated with the natural product. These results are in line with our hypothesis that (-)-hopeaphenol has low cell permeability. As for *Y. pseudotuberculosis* the data indicates that (-)-hopeaphenol binds covalently to *C. trachomatis*, inhibits successful infection and possibly reduces their growth inside the eukaryotic cell. (-)-Hopeaphenol is a selective and irreversible T3SS inhibitor with activity in eukaryotic whole cell infection assays with *Y. pseudotuberculosis, P. aeruginosa*, and *C. trachomatis* signifying plants as a valuable source for natural products that block virulence systems in human pathogens.

## Experimental

### YopE reporter-gene and YopH phosphatase assays

Luciferase analysis was performed essentially as described before [6,26]. The *Yersinia pseudotubeculosis* serotype III strain YPIII(pIB102) *yopE-luxAB* (Table 1) was grown over night in LB supplemented with Km, diluted to OD_600_ 0.1 in Ca^2+^ depleted LB and dispensed in 96-well plates (Nunc^TM^, flat bottom, white) with 100 µL / well. Compound was added to a final concentration between 1 and 100 µM. Final DMSO concentration did not exceed 1 %. The plates were incubated on a rotary shaker for 1 h at 26 °C followed by 2 h at 37 °C. The plates were allowed to adjust to room temperature on the bench for 2 h before 50 µL of decanal in water (10 µL / 100 mL) was added. After 4 minutes chemiluminescence was detected by a microplate reader (TECAN Infinite M200, gain 150, integration time 20 ms). An enzymatic YopH phosphatase assay was performed in parallel to the luciferase analysis. After 1 h incubation at 26 °C and 2 h at 37 °C the bacterial suspension (10 µL per well) was transferred to new 96-well plates (Nunc^TM^, flat bottom, transparent) containing the YopH substrate mixture (90 µL, 25 mM, p-nitro phenyl phosphate, 40 mM 2-(N-morpholino)ethanesulfonic acid, pH 5.0, and 1.6 mM dithiothreitol in water). The plates were incubated at 37 °C for 15 min. To quench the reaction NaOH (20 µL, 1 M) was added to each well. The absorbance was measured at 405 nm in a microplate reader (TECAN Infinite M200). 

**Table 1:** Characteristics and references of bacterial strains used in this study.

Bacterial strain	Characteristics	Reference
*Yersinia pseudotuberculosis*		
YPIII(pIB102)	Wild-type	[66]
YPIII(pIB102) *yopE-luxAB*	Luciferase reporter under the control of YopE promotor	[67]
YPIII(pIB604) Δ*yopB*	Translocation deficient	[68]
YPIII(pIB102) *yopE-bla*	β-lactamase translationally fused to YopE	[52]
*Pseudomonas aeruginosa*		
PAK	Wild-type	D. Bradley
PAK *exsA*::Ω	T3SS defective	[69]
*Chlamydia trachomatis*	serovar L2	VR-902B, ATCC

### Western blot analysis of total culture and supernatant samples from *Y. pseudotuberculosis*

Dose-response experiment: An overnight culture of *Yersinia pseudotuberculosis* YPIII(pIB102) (Table 1) was diluted 1:20 in Ca^2+^ depleted LB. The test compound was added to final concentration between 1.6 - 100 µM. The cultures were incubated for 1 h at 26 °C followed by 3 h at 37 °C. The cultures were centrifuged and the supernatant was mixed with sample buffer and loaded on to a 12 % SDS polyacrylamide gel. The gel was blotted onto a PVDF membrane. Blots were probed with polyclonal rabbit anti Yop-antiserum and polyclonal goat anti-rabbit immunoglobulins/HRP (Dako Denmark). Chemiluminiscense was generated using Millipore Immobilon^TM^ Western chemiluminescent HRP substrate and detected with CCD camera. Reversibility experiment: An overnight culture of YPIII(pIB102) was diluted 1:20 in LB supplemented with 2.5 mM CaCl_2_. Compound was added to a final concentration of 40 µM, control cultures received only DMSO. Cultures were first incubated at 26 °C for 30 min followed by 2 h at 37 °C for induction of Yop expression. To study the reversibility of the compound the samples was centrifuged and the pellet washed with LB once. Then LB complemented with 20 mM MgCl_2_ and 5 mM EGTA for Ca^2+^ depletion or 2.5 mM CaCl_2_, with 40 µM compound or DMSO was added followed by incubation at 37 °C for 45 min. The supernatant was mixed with sample buffer and the rest of the experiment was the same as described for the dose-response experiment. Time study: Analyses of the reporter-gene signal with compound added at different time points of the T3SS induction. Overnight culture of YPIII(pIB102) *yopE-luxAB* was diluted to OD_600_ 0.1 and dispensed in 96-well plate with 100 µL in each well. Compound (1 µL, 5 mM) was added at different time points t = 0, 30, 60, 90, 120, 150, and 180 minutes, where t = 60 is at the temperature shift. Incubation and measurements was the same as for luciferase experiments. 

### Inhibition of effector protein translocation in *Y. pseudotuberculosis*

The experiments were performed essentially as described previously [52,65]. HeLa 229 cells (ATCC**^®^** CCL-2.1^TM^) were seeded into 96-well plates (Nunc^TM^, flat bottom, transparent), 100 µL 1x10^5^ cells / mL one day before translocation experiment. Overnight cultures of YPIII(pIB102) *yopE-bla* and YPIII(pIB604) *yopE-bla* (Table 1) was diluted 1:10 in Ca^2+^ depleted LB and grown 1 h at 26 °C followed by 2 h at 37 °C. (-)-Hopeaphenol was added to the HeLa 229 cells at the same time as infected with MOI 50. The cells were infected for 1 h at 37 °C 5 % CO_2_ followed by 15 min at room temperature. The cells were loaded with LiveBLAzer^TM^ FRET - B/G loading kit with CCF4-AM protocol from Invitrogen according to the manufacture’s instructions. Fluorescence was measured in a microplate reader (Synergy H4 Hybrid Reader, BioTek^®^). For microscope pictures HeLa 229 cells were seeded into 35 mm glass-bottom microwell dishes (MatTek) 2 mL, 1.5x10^5^ cells/mL the day before translocation experiments. Translocation experiments and labeling was undertaken as written above. 

### Western blot analysis of total culture and supernatant samples from *P. aeruginosa*

Overnight cultures of *P. aeruginosa* (strains PAK and PAK *exsA*::Ω, a T3SS mutant, Table 1) in brain heart infusion medium (BHI; BD) were diluted 1:100 in either Ca^2+^ enriched (2.5 mM CaCl_2_) or Ca^2+^ depleted (5 mM EGTA and 10 mM MgCl_2_) BHI medium and (-)-hopeaphenol or 1 % DMSO (as a control) were added. The tubes were incubated for 3 h at 37 °C with shaking (250 rpm). The total and supernatant samples were prepared and run on NuPAGE 10 % Bis-Tris gel (Life Technologies) according to the manufacturer’s protocol. In brief, by mixing the samples with 4x NuPAGE LDS sample buffer (Life Technologies) and DTT, heating to 70 °C and loading onto the gel. ExoS protein was visualized using rabbit anti-ExoS serum and donkey anti-rabbit HRP-linked Ig (Amersham Bioscience) by ECL Western blot (GE Healthcare), which was performed according to standard procedures. Band intensities were analyzed in a FluorChem Q MultiImage III image system (Alpha Innotech).

### Infection of HeLa cells with *P. aeruginosa*

HeLa cells were seeded into flat-bottom 96-well plates (0.2 × 10^5^ cells/well) in DMEM (GlutaMAX –I, Gentamicin, FBS:Life Technologies) and incubated overnight at 37 °C 5 % CO_2_. Overnight cultures of *P. aeruginosa* in Luria-Broth (LB; Sigma-Aldrich) were diluted 1:2 in DMEM without phenol red (Life Technologies), supplemented with 3.97 mM l-alanyl-l-glutamine, and incubated for 1 h at 37 °C with shaking (250 rpm). The bacteria were then diluted to an OD_600_ of 0.0004. HeLa cells were washed (in DMEM without phenol red, supplemented with 10 % FBS and 3.97 mM l-alanyl-l-glutamine) and different concentrations of (-)-hopeaphenol were added to the plate, followed by addition of bacterial solution to a final OD_600_ of 0.0002. The cytotoxicity of (-)-hopeaphenol was tested on a plate with the same layout but with no addition of bacteria. The plates were incubated at 37 °C 5 % CO_2_ for 5 h and cell morphology was investigated using an inverted phase contrast microscope (Nikon Eclipse TE2000-S; Nikon) and photographs were taken using a Nikon Digital Sight DS-U2 camera (Nikon) and imaging software NIS-Elements (Nikon). 

### Infection experiments with *C. trachomatis*

For *Chlamydia* experiments, HeLa 229 cells were seeded into 96-well plates and 24-well plates with coverslips. Cells were infected the next day with 0.1 multiplicities of infection (MOI) of *C. trachomatis* serovar L2 (VR-902B, ATCC**^®^**) in Hank’s Balanced Salt Solution (HBSS). Infection was done in triplicates in 96-well plates and in duplicate in 24-well plates. To determine the minimum inhibitory concentration (-)-hopeaphenol was added 1 h after infection at concentrations ranging from 100 to 6 µM. For the pretreatment experiment either the cells or the *C. trachomatis* was treated for 1 h with indicated concentrations of (-)-hopeaphenol in room temperature. The (-)-hopeaphenol was removed from pretreated: host cells by changing the medium to HBSS containing DMSO treated *C. trachomatis*, and from pretreated *C. trachomatis* by centrifuging the bacteria down at 21,000 × *g* removing the supernatant and suspending the bacteria in fresh HBSS. Controls were treated similarly with equal amount of DMSO added.

The 96-well plates were fixed with methanol 19 h post infection and stained with DAPI and in-house antichlamydial rabbit antibody labeled with secondary FITC anti-rabbit antibody. The infection was analyzed using ArrayScan VTI HCA Reader (Thermo Fisher Scientific Inc. Waltham. MA), which automatically generated photomicrographs using 20 × objective. The *Chlamydia* inclusions were calculated with spot detection method included in the ArrayScan software.

The 24-well plates were fixed with methanol 48 h post infection and labeled similarly to 96-well plates. The micrographs were taken with Confocal Nikon 90i Eclipse microscope equipped with C1 Plus confocal (Nikon Instruments Europe B.V., Amsterdam, Netherlands) and EZ-C1 3.91 software. Exaction wavelengths were 405 and 488 nm and emissions were detected at 430/435 and 515/530 nm. The picture content was clarified by adjusting the curves of individual micrographs in Adobe Photoshop C5 Extended 12.0.4 (Adobe Systems Inc., San Jose, CA). Adjustments were done in similar manner for each micrograph.

### Treatment of five gram-negative and five gram-positive bacteria

Overnight cultures of the gram-positive bacteria *Micrococcus luteus, Staphylococcus epidermis, Staphylococcus aeureus, Bacillus subtilis*, and *Enterococcus faecalis* and the gram-negative bacteria *Y. pseudotuberculosis P. aeruginosa, Proteus mirabilis, Klebsiella pneumonia*, and *E. coli K12* were diluted to OD_600_ 0.1 in 96-well plates 25, 50, and 100 µM (-)-hopeaphenol final concentration was added to the wells in triplicate. Plates were incubated at 37 °C and samples were mixed before OD_600_ was checked at start, after 8 and 24 h. 
